# An Indian Mission and "The Hospital."

**Published:** 1919-03-22

**Authors:** E. M. Newman

**Affiliations:** Missionary in Charge, C. E. Z. Mission Dispensary, Rainawari, Srinagar—Kashmir, N. India


### An Indian Mission and "The Hospital."

*To the Editor of* The Hospital.

Dear Sir,—Readers of The Hospital may remember the account, published in your issue of August 24 last, of our hospital having been destroyed by fire. The result was a donation of £5 5s. from Nursing Sister Lucy J——. You will be glad to hear so far we have got the roof on the new building, and there will be three staircases instead of one, as in the old buildings, also space for four more beds.

Three hundred panes of glass will be needed, at 1s. per pane, and 5s. per 100 the man's wages for fixing in the glass. Tintacks and putty for same 7s. for 150 panes.

Dear fellow-workers who read The Hospital, can you help in this matter of glass? Postal orders of 1s. will be thankfully received, and if four were to give threepence each and send a postal order of 1s., it can be changed at the British Post Office at Srinagar. If you could see the sickness and poverty of this land I feel sure we should soon have the glass.

We got 3,000 bricks with the £5 5s. from Sister Lucy J——, so if you can count you will see what a large piece of wall she has built.

Believe me, dear fellow-workers in relief of suffering and pain, here is a people so poor that in dear old England you cannot realise their poverty.

C. E. Z. Mission Dispensary, Rainawari, Srinagar— Kashmir, N. India, February 1, 1919.

(Miss) E. M. Newman. Missionary in Charge.

